# MiR-486-5p Suppresses Proliferation and Migration of Hepatocellular Carcinoma Cells through Downregulation of the E3 Ubiquitin Ligase CBL

**DOI:** 10.1155/2019/2732057

**Published:** 2019-12-28

**Authors:** Jia He, Bin Xiao, Xiaoyan Li, Yongyin He, Linhai Li, Zhaohui Sun

**Affiliations:** ^1^Department of Laboratory Medicine, General Hospital of Southern Theatre Command of PLA, Guangzhou, Guangdong 510010, China; ^2^Genetic Laboratory, Maternal and Child Health Care Hospital of Baiyun District, Guangzhou, Guangdong 510010, China; ^3^Department of Laboratory Medicine, Shunde Affiliated Hospital of Guangzhou Medical University, Foshan, Guangdong 510010, China

## Abstract

MicroRNAs have been broadly implicated in cancer, but precise functions and mechanisms in carcinogenesis vary among cancer types and in many cases remain poorly understood. Hepatocellular carcinoma (HCC) is among the most frequent and lethal cancers. The aim of the present study was to investigate the role of miR-486-5p in HCC and identify its specific target. MiR-486-5p was significantly downregulated in HCC tissues and cell lines compared with noncancerous tissues and, respectively, although expression level was not correlated with the degree of infiltration or tumor stage. However, miR-486-5p overexpression in HCC cells inhibited proliferation and migration as evidenced by CCK-8 cell counting, wound healing, and transwell assays, indicating that miR-486-5p is an HCC suppressor. We employed four miRNA databases to predict the target genes of miR-486-5p and verified retrieved genes using qPCR and western blotting. The E3 ubiquitin ligase CBL was significantly downregulated by miR-486-5p overexpression in HCC cell lines at both mRNA and protein level, and overexpression of CBL counteracted the inhibitory effects of miR-486-5p on HCC cell proliferation and migration. Moreover, CBL expression was negatively correlated with miR-486-5p expression in HCC tissues. Collectively, our results suggest that miR-486-5p may act as a tumor suppressor gene in HCC by downregulating CBL expression.

## 1. Introduction

Hepatocellular carcinoma (HCC) is the sixth most common cancer worldwide and the third leading cause of cancer-related mortality [1]. Although improvements in diagnostic techniques have increased early detection and decreased mortality over the past decade, the incidence of HCC continues to increase and overall outcomes remain poor, with 5-year overall survival (OS) rate of only 3%–5% [2]. Early diagnosis and treatment is critical for improved prognosis. Therefore, new targets for preventing the initiation and progression of HCC are urgently required.

MicroRNAs (miRNAs) are a class of 20–25 nt small RNAs that silence the transcription of specific genes participating in diverse physiological and pathological processes, including carcinogenesis [3]. Evidence is accumulating that miRNAs are dysregulated in various human cancers, including HCC. These dysregulated miRNAs are often involved in processes relevant to tumorigenesis, tumor growth, and metastasis, such as cell proliferation, apoptosis, angiogenesis, and migration, thereby acting as oncogenes or tumor suppressors [4–7]. MiR-486-5p, encoded by the 40th intron of the ankyrin-1 gene, was first discovered in fetal liver and subsequently implicated in the development of many diseases including tumors. It has been reported that miR-486 relieves particulate matter-induced injury of human lung alveolar epithelial A549 cells by targeting PTEN and FOXO1 [8]. Mimics of miR-486-5p also inhibited the progression of colorectal cancer (CRC) by inhibiting the AKT signaling pathway through PIK3R1 downregulation [9]. However, the biological functions and downstream targets of miR-486-5p in HCC have remained elusive.

CBL was discovered as a cellular homologue of the v-Cbl oncogene [10]. The CBL family is composed of Cbl, Cbl-b, and Cbl-c/Cbl-3, all of which structurally resemble E3 ubiquitin ligases. Recent studies have shown that E3 ubiquitin ligases regulate the development of neuropathic pain by attenuating the production of IL-2 [11]. CBL also regulates the proliferation, differentiation, and survival of human mesenchymal-derived osteoblasts [12]. Silencing Cbl-b expression in breast cancer cells enhanced the risk of lung metastasis in nude mice, and it was concluded that Cbl-b reduces RANK protein expression and inhibits RANKL-induced breast cancer cell migration through negative regulation of the Src-AKT/ERK pathway [13]. Therefore, Cbl appears to have multiple and often divergent effects on different cancer types, presumably by interacting with distinct partners or due to differential regulation by upstream factors, possibly including miRNAs.

In the present study, the functions of miR-486-5p in HCC cells were explored. Results demonstrate that miR-486-5p inhibits the proliferation and migration of HCC cells through downregulation of CBL. The miR-486-5p-CBL regulatory pathway may thus be a promising therapeutic target for the treatment of HCC.

## 2. Results

### 2.1. Downregulation of MiR-486-5p in HCC Tissues

**Figure 1:**
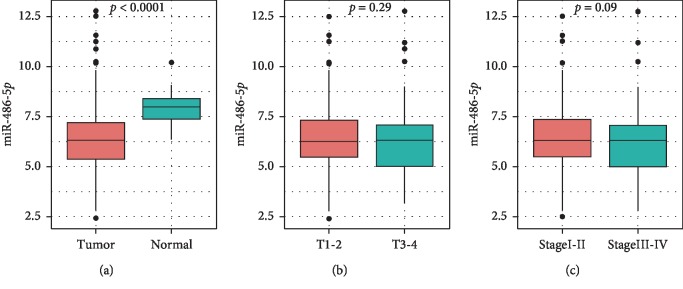
Expression of miR-486-5p is nonspecifically downregulated in hepatocellular carcinoma (HCC) tissue. (a) Expression of miR-486-5p in HCC and adjacent tissues. (b) Expression of miR-486-5p in HCC with different degrees of infiltration. (c) Expression of miR-486-5p in different stages of HCC.

The miRNA Seq data, mRNA Seq data, and corresponding HCC clinical data were downloaded from the TCGA database (http://tcga-data.nci.nih.gov/). A total of 422 samples were included in the miRNA Seq data, 372 of which were tumor samples and the remaining 50 normal tumor-adjacent samples. We found that miR-486-5p was downregulated in tumor samples compared with tumor-adjacent samples (*P* < 0.0001; Figure 1(a)). We then investigated the expression of miR-486-5p in different tumor stages and various degrees of infiltration from the clinical data but found no significant difference between T1-2 (*n* = 279) and T3-4 (*n* = 94) (*P*=0.29; Figure 1(b)) or between Stage I-II (*n* = 261) and Stage III-IV (*n* = 91) (*P*=0.09; Figure 1(c)).

### 2.2. Overexpression of MiR-486-5p Inhibits HCC Cell Proliferation and Migration

**Figure 2:**
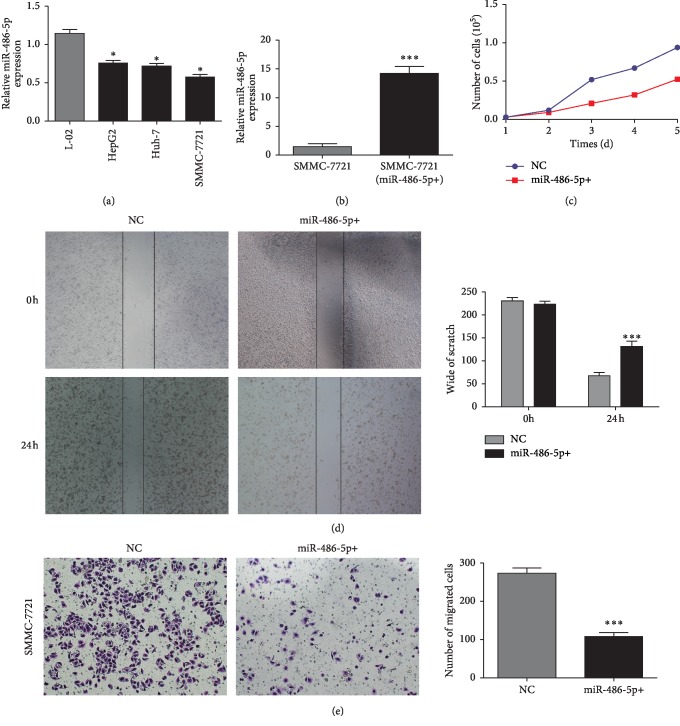
Overexpression of miR-486-5p inhibited the proliferation and migration of HCC cells. (a) Expression of miR-486-5p in normal liver cells (L-02) and HCC cell lines (HepG2, Huh-7, and SMMC-7721). (b) Expression of miR-486-5p in SMMC-7721 cells transfected with an overexpression vector compared with control SMMC-7721 cells. (c) CCK-8 assay showing reduced proliferation of SMMC-7721 HCC cells overexpressing miR-486-5p compared with control SMMC-7721 cells. (d) Wound-healing assay showing that miR-486-5p overexpression reduced the migratory capacity of SMMC-7721 cells. (e) Transwell assay confirming reduced migratory ability of SMMC-7721 HCC cells overexpressing miR-486-5p. (^*∗*^*P* < 0.05; ^*∗∗∗*^*P* < 0.001, Student's *t*-test).

To examine miR-486-5p functions in tumorigenesis, we first compared miR-486-5p expression in three HCC cell lines (SMMC-7721, HepG2, and Huh-7) with noncancerous cells by qRT-PCR. MiR-486-5p was significantly downregulated in all three HCC lines compared with the noncancerous human fetal hepatocyte cell line L-02 (*P* < 0.05) (Figure 2(a)), suggesting that loss of miR-486-5p function (target gene regulation) is involved in hepatic tumorigenesis. We chose SMMC-7721, the HCC cell exhibiting the lowest miR-486-5p expression, to construct a recombinant cell line stably overexpressing miR-486-5p (Figure 2(b)) and conducted CCK-8 assays to examine the effects of miR-486-5p on cell proliferation and migration. As shown in Figure 2(d), SMMC-7721 cells overexpressing miR-486-5p proliferated more slowly than control SMMC-7721 (*P* < 0.001). Wound-healing assay and transwell assay also revealed that miR-486-5p overexpression significantly reduced the migratory capacity of SMMC-7721 cells (Figure 2(e)) (*P* < 0.001). Collectively, these results suggest that miR-486-5p suppresses tumor activity by reducing growth and metastasis.

### 2.3. CBL Is a Target of MiR-486-5p

**Figure 3:**
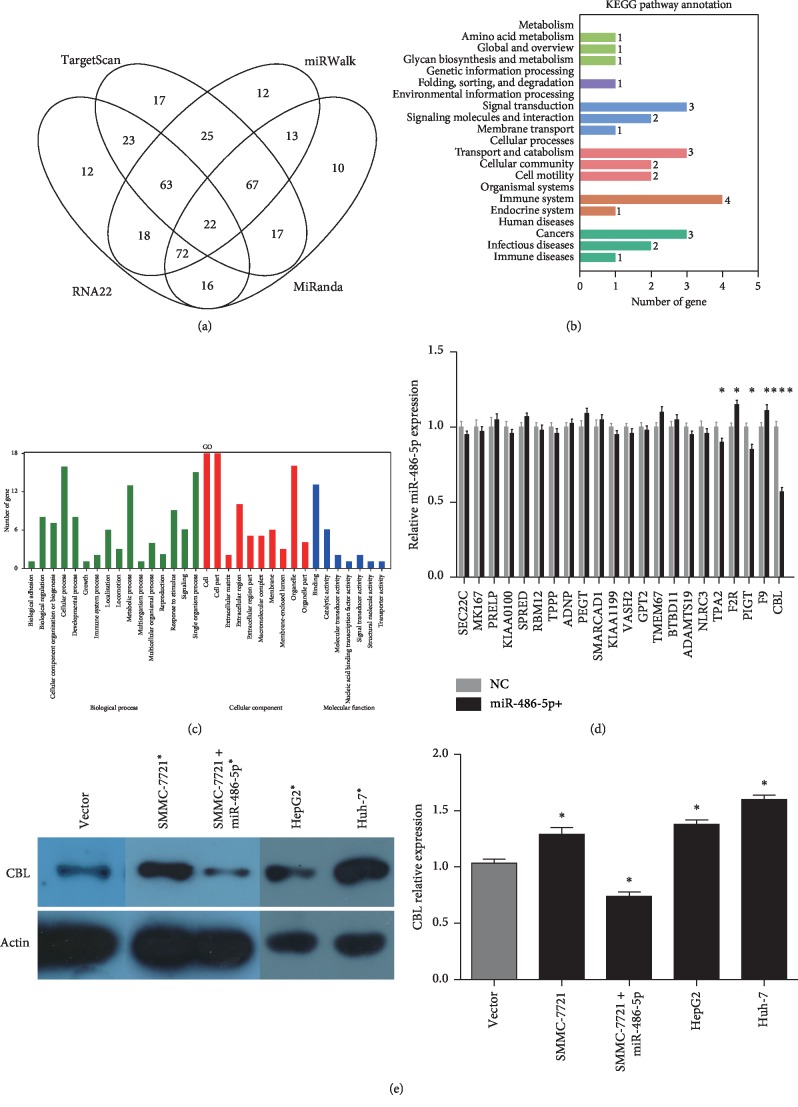
CBL is a downstream target of miR-486-5p. (a) Venn diagram constructed using four miRNA databases showing genes that may be regulated by miR-486-5p. (b) KEGG analysis of 22 potential targets of miR-486-5p. (c) GO analysis of these 22 potential targets of miR-486-5p. (d) Expression of these 22 target genes in control SMMC-7721 hepatoma cells and SMMC-7721 cells overexpressing miR-486-5p as measured by qPCR. (e) Expression of CBL in HCC cell lines, including SMMC-7721 cells overexpressing miR-486-5p, measured by western blot. (^*∗*^*P* < 0.05; ^*∗∗∗*^*P* < 0.05, Student's *t*-test).

The mechanisms underlying inhibition of tumor growth by miR-486-5p were then examined. We predicted the target genes of miR-486-5p using four miRNA databases: TargetScan, miRWalk, RNA22, and miRanda. A total of 22 candidate target genes were found by all 4 databases (Figure 3(a)). According to KEGG pathway annotation, these 22 genes were enriched in signal transduction and immune system categories (Figure 3(b)). GO analysis indicated that these 22 genes were widely distributed across a variety of cellular component groups and biological processes (Figure 3(c)). To verify whether the expression levels of these 22 genes could be regulated by miR-486-5p, mRNA expression levels were measured by qPCR in SMMC-7721 cells stably overexpressing miR-486-5p. As shown in Figure 3(d), the expression of CBL was downregulated to the greatest extent by miR-486-5p (*P* < 0.001) among the 22 genes. The expression levels of TPA2 and PIGT were also inhibited by miR-486-5p but to a lesser extent, and F2R and F9 were upregulated by miR-486-5p overexpression. We thus focused on CBL as a potential mediator of miR-486-5p effects on HCC cell line proliferation and migration. We first compared the expression of CBL among cell lines and found upregulation in HCC lines compared with control cells (*P* < 0.05) (Figure 3(e)). Conversely, CBL was downregulated in SMMC-7721 cells overexpressing miR-486-5p (*P* < 0.05), suggesting that miR-486-5p inhibits CBL expression (Figure 3(e)).

### 2.4. CBL Overexpression Counteracts the Effects of MiR-486-5p Overexpression on HCC Cell Proliferation and Migration

**Figure 4:**
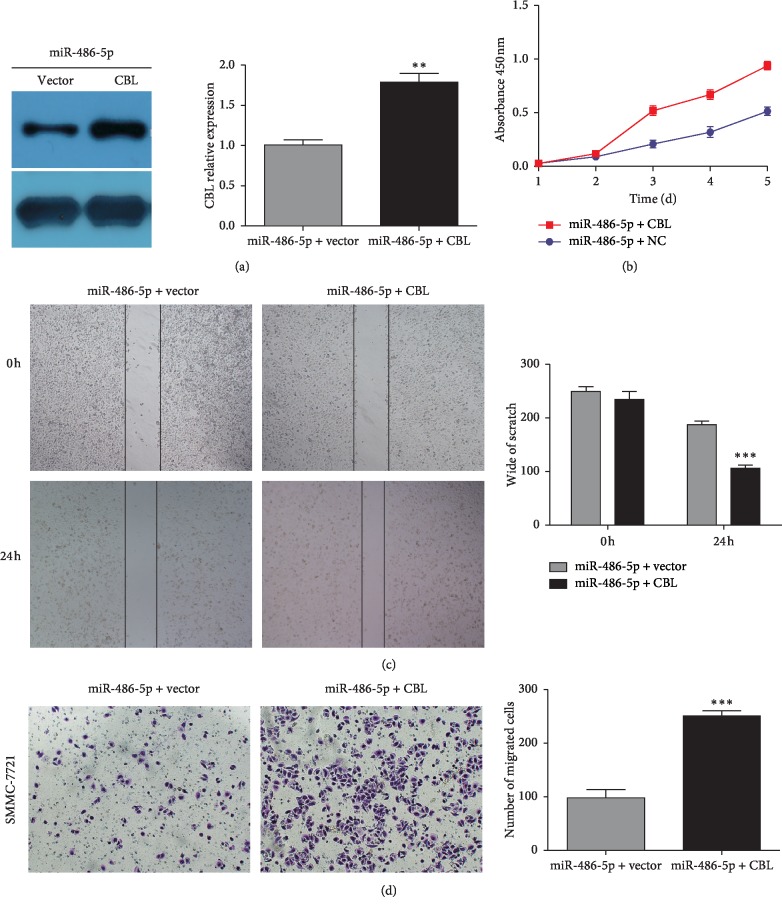
Overexpression of CBL counteracted the inhibitory effect of miR-486-5p on HCC cell proliferation and migration. (a) Characterization of a CBL-overexpressing cell line derived from miR-486-5p-overexpressing SMMC-7721 cells. (b) Overexpression of CBL enhanced SMMC-7721 cell proliferation. Overexpression of CBL enhanced SMMC-7721 cell migration in the wound-healing assay (c) and transwell assay (d) (^*∗*^*P* < 0.05; ^*∗∗*^*P* < 0.01; ^*∗∗∗*^*P* < 0.001, Student's *t*-test).

To examine whether miR-486-5p functions through CBL modulation, we established an HCC cell line overexpressing both CBL and miR-486-5p by transfection of miR-486-5p-overexpressing SMMC-7721 cells with a CBL expression plasmid. CBL overexpression was verified by western blot (Figure 4(a), *P* < 0.01). Overexpression of CBL significantly enhanced the proliferation of the line stably overexpressing miR-486-5p (Figure 4(b), *P* < 0.01). Overexpression of CBL also increased the migration of HCC cells overexpressing miR-486-5p in wound healing and transwell migration assays (Figures 4(c), 4(d), *P* < 0.01). Based on these results, we conclude that miR-486-5p exerts its biological functions at least partly through downregulation of CBL.

### 2.5. Prognostic Values of MiR-486-5p and CBL

**Figure 5:**
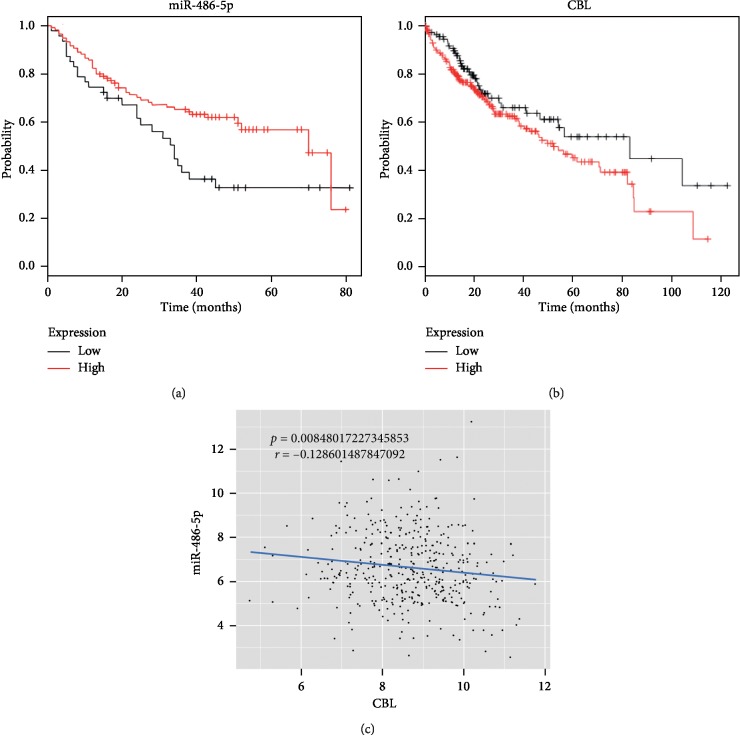
The prognostic value of miR-486-5p and CBL expression levels for HCC outcome. Kaplan–Meier analyses showing the correlation of miR-486-5p (a) and CBL (b) with HCC prognoses. (c) The inverse correlation between miR-486-5p and CBL expression levels.

Finally, we analyzed the correlations of miR-486-5p and CBL expression levels with HCC prognosis. Higher miR-486-5p expression predicted better survival probability (Figure 5(a)), while higher CBL expression predicted poorer prognosis (Figure 5(b)). Moreover, miR-486-5p was negatively correlated with CBL expression in clinical HCC patient samples (Figure 5(c)). Thus, interventions enhancing miR-486-5p and (or) reducing CBL may improve HCC prognosis.

## 3. Discussion

Although the overall survival rate of HCC patients has increased in recent years, metastasis and recurrence are still common. Factors controlling these processes are thus potentially promising targets for life-extending therapies. MicroRNAs are novel molecular markers for many diseases and are gaining increased importance for diagnosis and treatment. It is expected that miRNAs will also be identified that contribute to HCC and thus provide new targets for screening and individualized HCC treatment. In the present study, we demonstrate that miR-486-5p inhibits the proliferation and migration of HCC cells, at least in part by downregulating expression and activity of the E3 ubiquitin ligase CBL.

The miR-486 encoding site on human chromosome 8 yields three alternative species, miR-486-3p, miR-486-5p, and miR-486, all of which have been shown to be aberrantly expressed in human cancers [14]. Thus, miR-486 may serve as a biological marker for diagnosis [15]. MiR-486 has been shown to interfere with gastric cancer cell apoptosis by regulating PTEN and PIM-1 [16, 17], and elevated expression may have a more general antiapoptotic effect as it is associated with reduced expression of the tumor suppressor p53 and the apoptosis effector caspase-3 [18]. Conversely, miR-486 was found to suppress the growth and metastasis of esophageal cancer by targeting CDK4/BCAS2 [19]. Low miR-486 expression has been observed in esophageal squamous cell carcinoma and gastric adenocarcinoma, suggesting utility as an independent marker to evaluate prognosis [20–22]. MiR-486-5p may also act as a tumor suppressor in lung and gastric cancers, but other studies have shown that miR-486-5p plays a procarcinogenic role in Down's syndrome-associated myeloid leukemia and chronic myelogenous leukemia [20]. Thus, miR-486 exhibits complex and sometimes opposing effects on malignancy.

In the present study, we found lower miR-486-5p expression in HCC tissues and HCC cell lines compared with surrounding noncancerous tissues and noncancerous hepatocytes, respectively, consistent with a tumor-suppressor function. Indeed, overexpression inhibited both the proliferation and migration of HCC cells, suggesting that miR-486-5p upregulation (or downregulation of its target) may be a novel treatment strategy.

CBL is an E3 ubiquitin ligase and multifunctional adaptor that regulates cell growth, invasion, apoptosis, and angiogenesis in multiple malignancies [23]. For example, CBL generally targets receptor tyrosine kinases and other nonreceptor tyrosine kinases, such as Src family kinases, and mutations in the components of this pathway may result in tumorigenesis [24]. Previous studies have confirmed that Cbl increases the chemosensitivity of gastric cancer cells by enhancing the epidermal growth factor receptor (EGFR) and mitochondrial signaling pathways [25]. High CBL expression has actually been recognized as linked with the development of several types of cancer, including gliomas, gastric carcinoma, colorectal cancer, prostate cancer, breast cancer and non-small-cell lung cancer [26]. A previous study has shown that CBL might be a prognostic indicator of HCC [27]; however, the biological effect of CBL in HCC has not been reported. In the current study, CBL was demonstrated as a downstream target of miR-486-5p, and CBL overexpression reversed the antitumor functions of miR-486-5p in HCC.

In summary, we found that higher miR-486-5p is associated with better prognosis of HCC and that overexpression can inhibit HCC cell proliferation and migration *in vitro*. These effects may stem from downregulation of its target CBL. These findings provide a targeted treatment strategy for HCC.

## 4. Materials and Methods

### 4.1. Cell Culture

The SMMC-7721, HepG2, and Huh-7 HCC cell lines and the control L-02 cell line were purchased from the Cell Bank of the Chinese Academy of Sciences, Shanghai Institutes for Biological Sciences (Shanghai, China). Cell lines were cultured in Roswell Park Memorial Institute (RPMI) 1640 medium (Life Technologies, Carlsbad, CA, USA) supplemented with 10% fetal bovine serum (FBS, Invitrogen, Carlsbad, CA, USA) and 1% penicillin/streptomycin (Sigma-Aldrich, St. Louis, MO, USA) at 37°C under a 5% CO_2_ atmosphere.

### 4.2. MicroRNA Target Prediction

Bioinformatics prediction of miRNA-486-5p target genes and specific miRNA binding sites was performed using a combination of four databases: TargetScan version 7.1 (http://www.targetscan.com), miRanda (http://www.microrna.org), miRWalk version 2.0 (http://zmf.umm.uni-heidelberg.de/apps/zmf/mirwalk2/custom.html), and RNA22v2 (http://www.mybiosoftware.com/rna22-v2-microrna-target-detection.html). Target genes were selected according to their *P* values.

### 4.3. Cell Proliferation Assay

Cell proliferation was evaluated using a CCK-8 kit according to the manufacturer's instructions (Dojindo, Japan). The assay relies on the metabolism of the tetrazolium salt WST-8 (2-(2-methoxy-4-nitrophenyl)-3-(4-nitrophenyl)-5-(2,4-disulfide benzene)-2H-tetrazole monosodium salt) by viable cells. Cells were plated into 96-well culture plates at an optimal density of 5 × 103 cells/ml in 100 *μ*l culture medium. After 24 h, WST solution was added (10 *μ*l/well) to each well and incubated at 37°C for 3 h. The optical density of each sample well was immediately measured at 450 nm using a microplate reader (Bio-Rad, Hercules, CA, USA).

### 4.4. Wound-Healing Assay

When cells were 70%–80% confluent, a scratch was created across the center of the culture plate and the cells were allowed to migrate into the wound for 28 h. Images were captured under an inverted microscope at 100x magnification. The gap distance was evaluated using TScratch v7.8.

### 4.5. Transwell Assay

For the transwell migration assay, 7 × 104 cells in 200 *μ*l serum-free Dulbecco's modified Eagles Medium (DMEM, Life Technologies, USA) were added to the upper chamber of two-chamber culture inserts separated by 8 *μ*m microporous filters without extracellular matrix coating (Becton Dickinson Labware, Bedford, MA, USA). DMEM containing 10% FBS was added to the bottom chamber. After 24 h of incubation, migrated cells on the lower surface of the filter were fixed, stained, and examined under light microscopy. The number of migrated cells in three random optical fields (×100 magnification) from triplicate filters was averaged.

### 4.6. Isolation of an HCC Cell Line Stably Overexpressing MiR-486-5p

PLVX-puro or PLVX-miR-486-5p was cotransfected along with pMD2.G and psPAX2 into HEK-293T cells for 48 h. The recombinant viruses were subsequently collected and added to SMMC-7721 cells, which were then cultured with 8 *μ*g/ml polybrene for 24 h. Stably infected cells were selected by treatment with 1 *μ*g/ml puromycin for two weeks.

### 4.7. RNA Isolation and Quantitative (q) RT-PCR

Total RNA was extracted from the cultured cells using Trizol Reagent according to the manufacturer's protocol (QIAGEN, Germany). To analyze the expression of miR-486-5p, reverse transcription and stem-loop RT-PCR were performed using TaqMan MicroRNA assays (Applied Biosystems, Foster City, CA, USA), and PCR products were amplified using a TaqMan Universal PCR Master Mix (Applied Biosystems). Relative expression of miR-486-5p was calculated using the 2-∆∆CT method and normalized to the expression of U6. All experiments were performed in triplicate.

### 4.8. Western Blot Analysis

Cells and tissues were lysed for 30 min in ice-cold RIPA lysis buffer (10 mM Tris (pH 8.0), 150 mM NaCl, 1% Nonidet P-40, 0.1% sodium dodecyl sulfate (SDS), and 0.5% deoxycholate) supplemented with the protease inhibitor phenylmethylsulfonyl fluoride (PMSF). Lysate samples were centrifuged at 14,000×*g* for 30 min at 4°C, and the supernatants were collected for SDS-polyacrylamide gel electrophoresis and western blotting according to standard protocols. Separated proteins were labeled with human and mouse anti-*β*-actin monoclonal antibodies (1 : 1000, Abcam, UK) and appropriate secondary antibodies (1 : 4000, Abcam, UK).

### 4.9. Statistical Analysis

Group means were compared by one-way ANOVA or Student's *t*-test with Welch's correction for unequal variance. All statistical analyses were conducted using GraphPad 5 software. A *P* < 0.05 (two tailed) was considered statistically significant.

## Data Availability

The data used to support the findings of this study are included within the article.
